# Supplementary material for: Genome editing is induced in a binary manner in single human cells
Source: iScience. 2022 Nov 17;25(12):105619. doi: 10.1016/j.isci.2022.105619 (PMC9722467; doi:10.1016/j.isci.2022.105619)
Supplement: Document S1. Figures S1–S13 and Tables S1–S9 [file mmc1.pdf]

iScience, Volume *25*

Supplemental information

Genome editing is induced in a binary manner in single human cells

Gou Takahashi, Daiki Kondo, Minato Maeda, Yuji Morishita, and Yuichiro Miyaoka

Supplemental Information

Genome Editing Is Induced in a Binary Manner in Single Human Cells

[1]Gou Takahashi, [1,2]Daiki Kondo, [1,2]Minato Maeda, [3]Yuji Morishita, and [1,2,4,5]Yuichiro Miyaoka*

[1]Regenerative Medicine Project, Tokyo Metropolitan Institute of Medical Science, Tokyo, Japan

[2]Graduate School of Medical and Dental Sciences, Tokyo Medical and Dental University, Tokyo, Japan

[3]On-chip Biotechnologies Co., Ltd., Tokyo, Japan.

[4]Graduate School of Humanities and Sciences, Ochanomizu University, Tokyo, Japan

[5]Lead contact

*Correspondence should be addressed to Yuichiro Miyaoka, miyaoka-yi@igakuken.or.jp

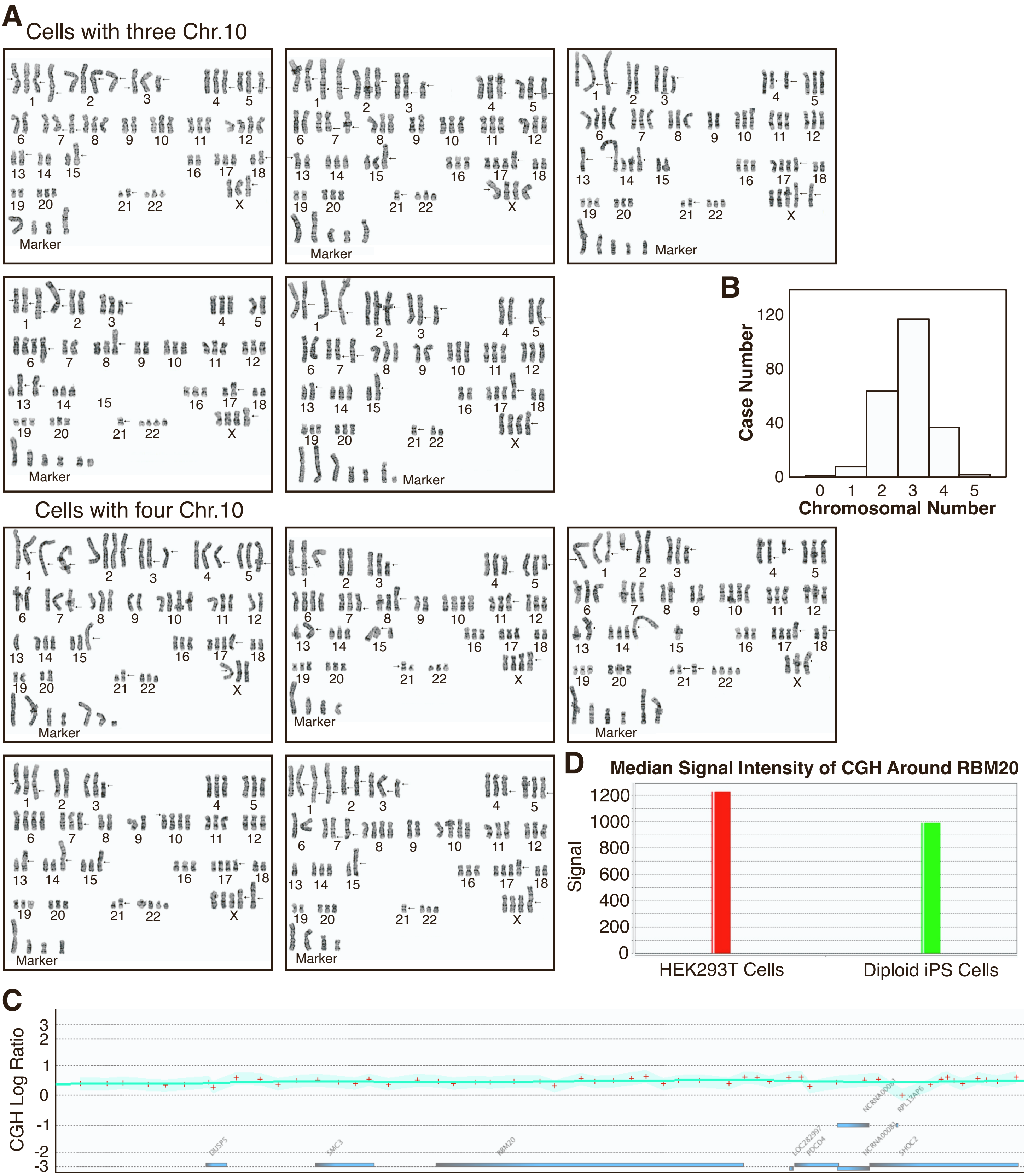

A
Cells with three Chr.10
Cells with four Chr.10
Marker
B
Case Number
Chromosomal Number
C
CGH Log Ratio
D
Median Signal Intensity of CGH Around RBM20
Signal
HEK293T Cells
Diploid iPS Cells

Figure S1. Karyotyping and a CGH analysis of HEK293T cells to estimate the copy number of RBM20, Related to Figure 1.

(A) Karyotypes of ten HEK293T cells. Five cells had three chromosome 10s, and the other five had four chromosome 10s. Please note that two of them are also shown in Figure 1B. (B) Case numbers observed for each chromosomal number in HEK293T cells. (C) Scattered plot of the relative CGH signal of HEK293T cells in comparison to diploid human iPS cells around the RBM20 gene. The relative CGH signals of HEK293T cells normalized by the CGH signals of iPS cells are represented by +. This panel shows a wider genomic region than Figure 1F. There were no detectable microduplications or microdeletions around the RBM20 locus. (D) The median signal intensity of CGH around RBM20. The median signal intensities of HEK293T cells and iPS cells in the genomic region shown in (C). By applying these values to calculate the CGH log ratio and applying it to the formula shown in Figure 1E, the copy number of RBM20 was calculated to be 3.55.

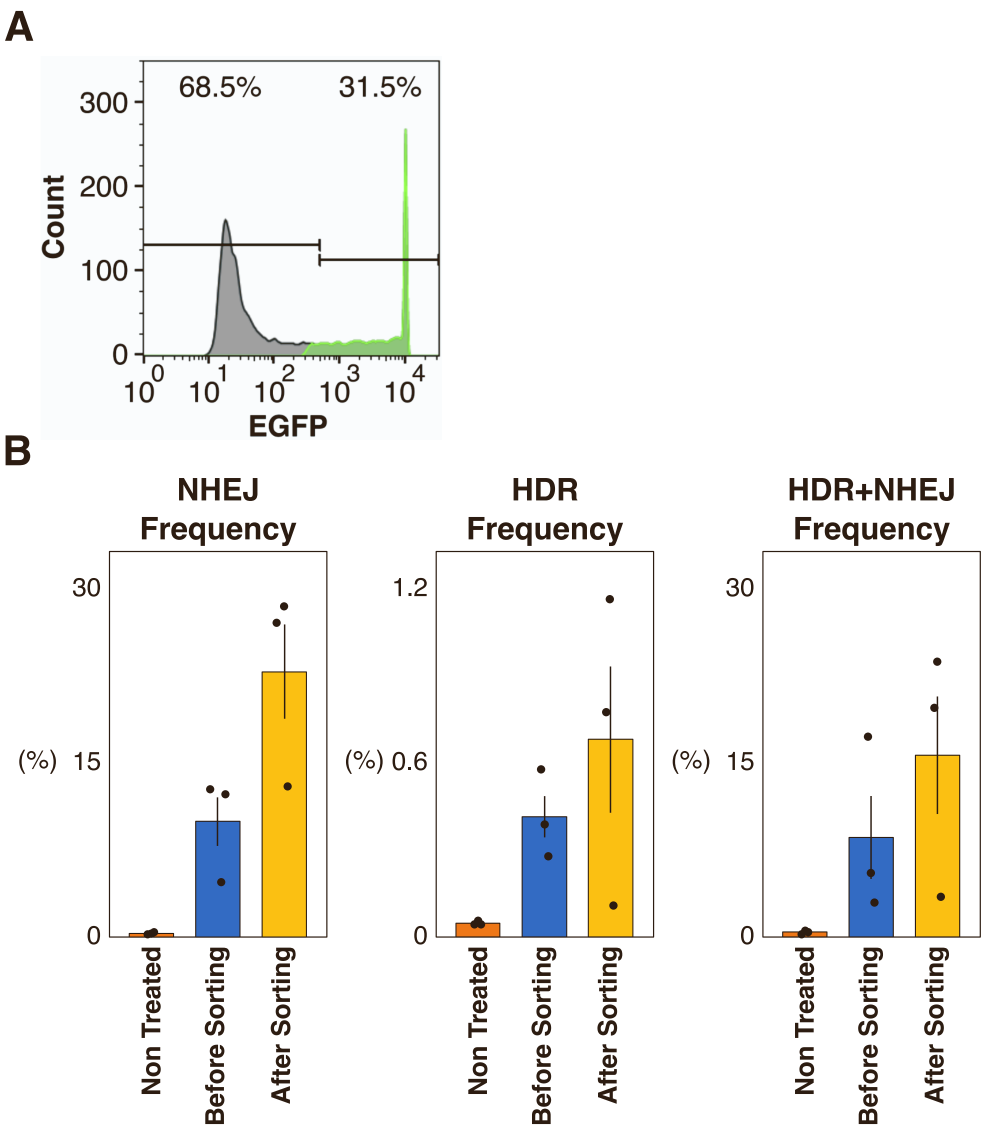

Figure S2. Sorting of EGFP-positive HEK293T cells, Related to Figure 2.

(A) Histogram showing the sorting gate of EGFP-positive HEK293T cells. (B) Frequencies of NHEJ, HDR, and HDR+NHEJ alleles before and after cell sorting. Cas9, gRNA targeting RBM20, and EGFP were co-expressed in HEK293T cells and EGFP-positive cells were sorted. The frequencies of NHEJ, HDR, and HDR+NHEJ alleles were analyzed by amplicon sequencing. Values ± S.E. are shown (n=3).

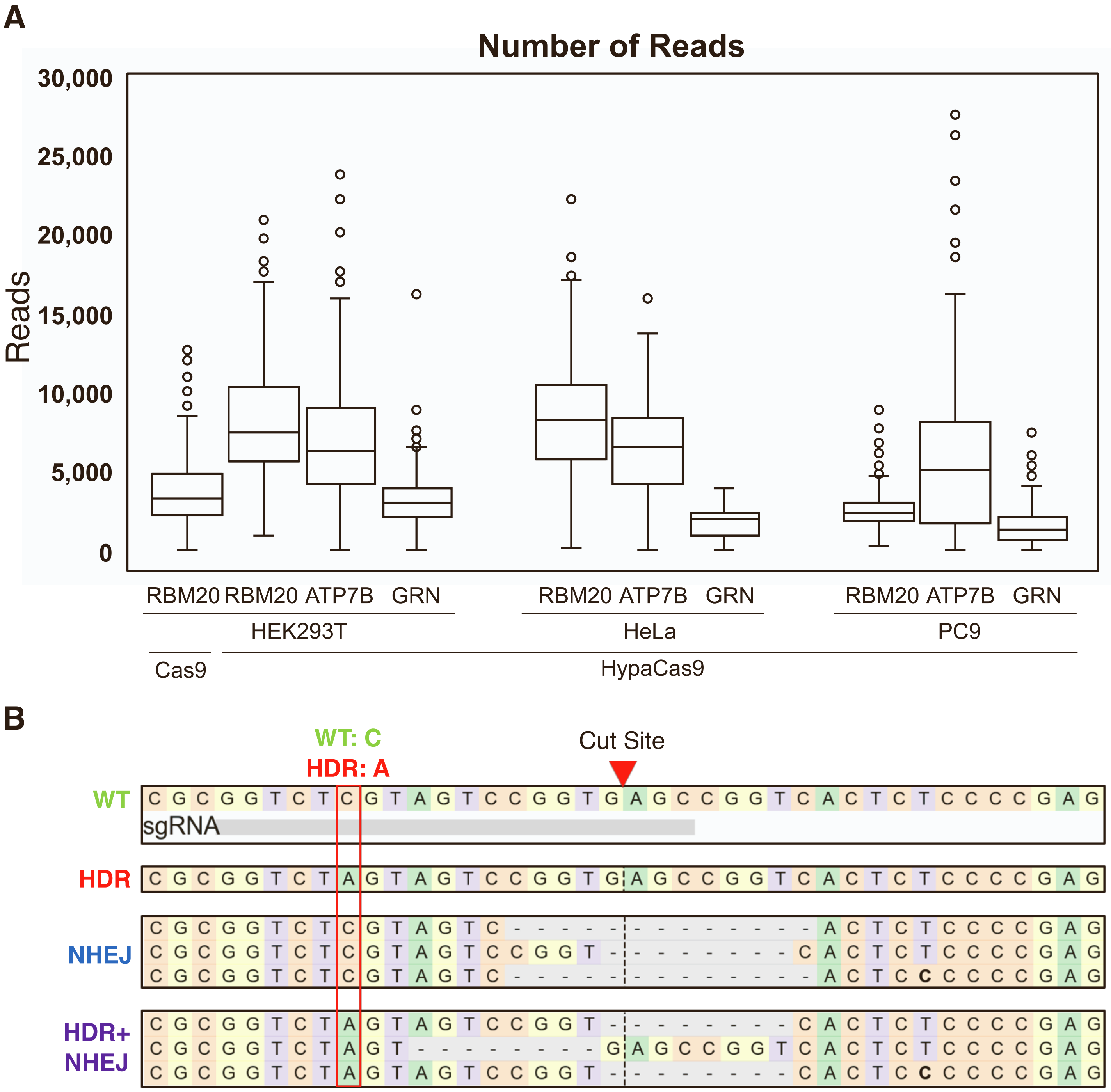

Figure S3. Amplicon sequencing of isolated clones, Related to Figure 2.

(A) The number of reads sequenced by amplicon sequencing in this study. (B) Example reads of amplicon sequencing of RBM20 targeting in HEK293T cells. The cut site and mutated site are shown.

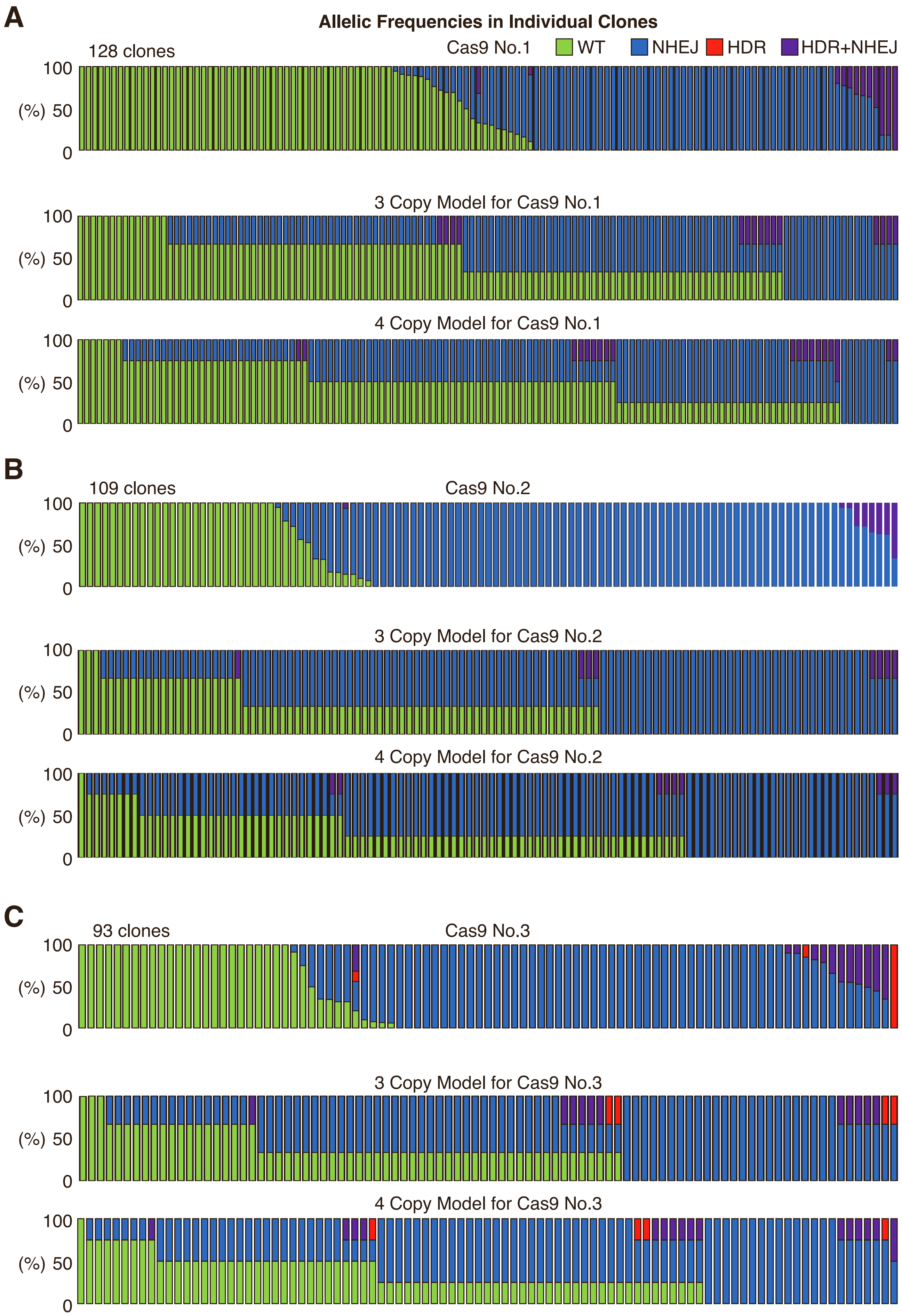
A
Allelic Frequencies in Individual Clones
128 clones
Cas9 No.1
WT
NHEJ
HDR
HDR+NHEJ
100
(%) 50
0
3 Copy Model for Cas9 No.1
4 Copy Model for Cas9 No.1
B
109 clones
Cas9 No.2
3 Copy Model for Cas9 No.2
4 Copy Model for Cas9 No.2
C
93 clones
Cas9 No.3
3 Copy Model for Cas9 No.3
4 Copy Model for Cas9 No.3

Figure S4. Models assuming random RBM20 R636S mutagenesis by Cas9 in HEK293T cells, Related to Figure 3.

(A-C) Models of the distribution of HEK293T cell clones with different genome editing outcomes by Cas9, if genome editing randomly occurred at the overall frequencies in HEK293T cells with three or four copies of RBM20 in experiment No. 1 (A), No. 2 (B), and No. 3 (C). The top row in each panel shows the observed distribution of the isolated HEK293T cell clones. Note that the original data and the 3 and 4 copy models for Cas9 No. 3 are also shown in Figure 3.

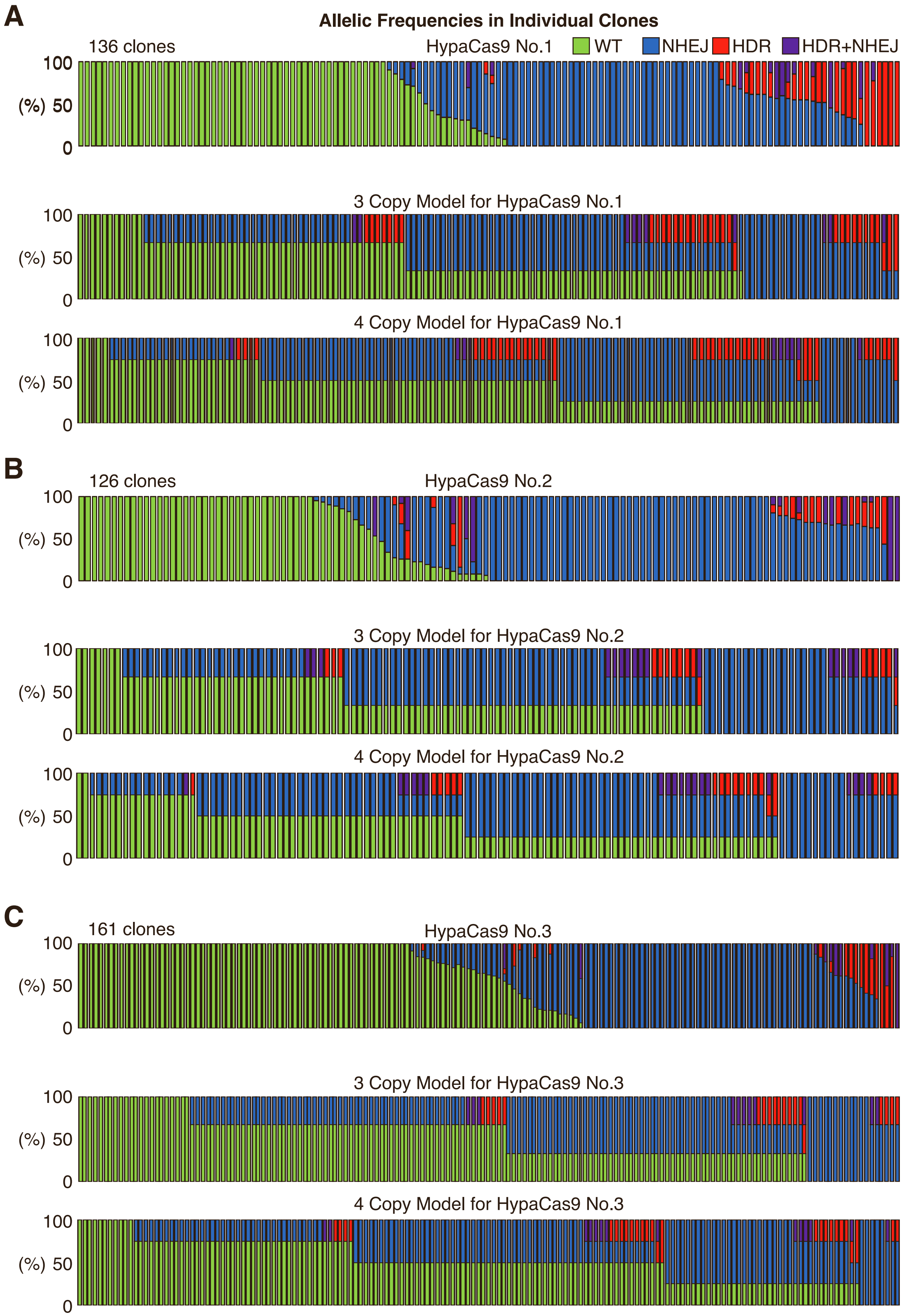
A
Allelic Frequencies in Individual Clones
136 clones
HypaCas9 No.1
WT
NHEJ
HDR
HDR+NHEJ
100
(%) 50
0
3 Copy Model for HypaCas9 No.1
4 Copy Model for HypaCas9 No.1
B
126 clones
HypaCas9 No.2
3 Copy Model for HypaCas9 No.2
4 Copy Model for HypaCas9 No.2
C
161 clones
HypaCas9 No.3
3 Copy Model for HypaCas9 No.3
4 Copy Model for HypaCas9 No.3

Figure S5. Models assuming random RBM20 R636S mutagenesis by HypaCas9 in HEK293T cells, Related to Figure 4.

(A-C) Models of the distribution of HEK293T cell clones with different genome editing outcomes by HypaCas9, if genome editing randomly occurred at the overall frequencies in HEK293T cells with three or four copies of RBM20 in experiment No. 1 (A), No. 2 (B), and No. 3 (C). The top row in each panel shows the observed distribution of the isolated HEK293T cell clones. Note that the original data and the 3 and 4 copy models for HypaCas9 No. 3 are also shown in Figure 4.

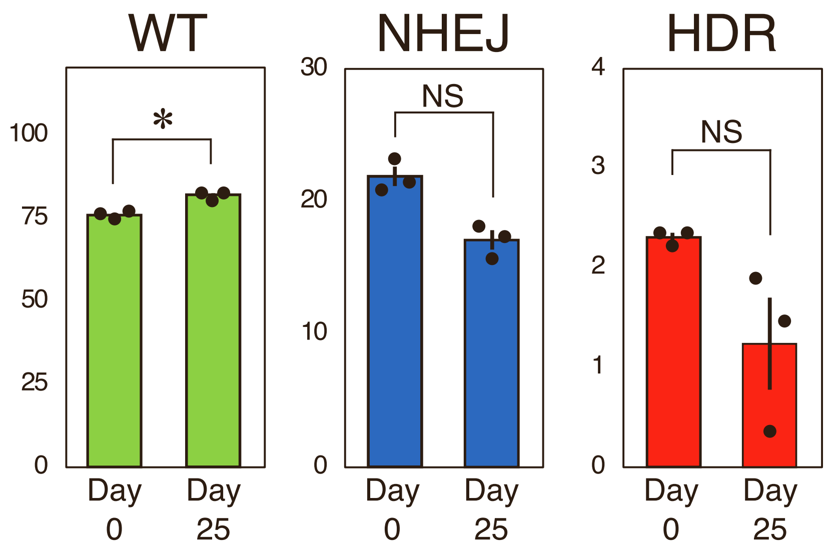

Figure S6. Change in frequencies of genome edited alleles over 25 days of culture, Related to Figure 4.

HEK293T cells were edited by using HypaCas9 to induce the R636S mutation in RBM20. Then, the cells were expanded for 25 days to see whether induced NHEJ or HDR would affect cell growth or survival. Over 25 days, the NHEJ and HDR frequencies did not significantly change, although the WT frequency slightly increased. These results indicate that growth or survival bias depending on genome editing outcomes was marginal in our analysis. Values ± S.E. are shown (n=3). Student's t-test was used to evaluate differences. *P<0.05. NS: not significantly different (P>0.1).

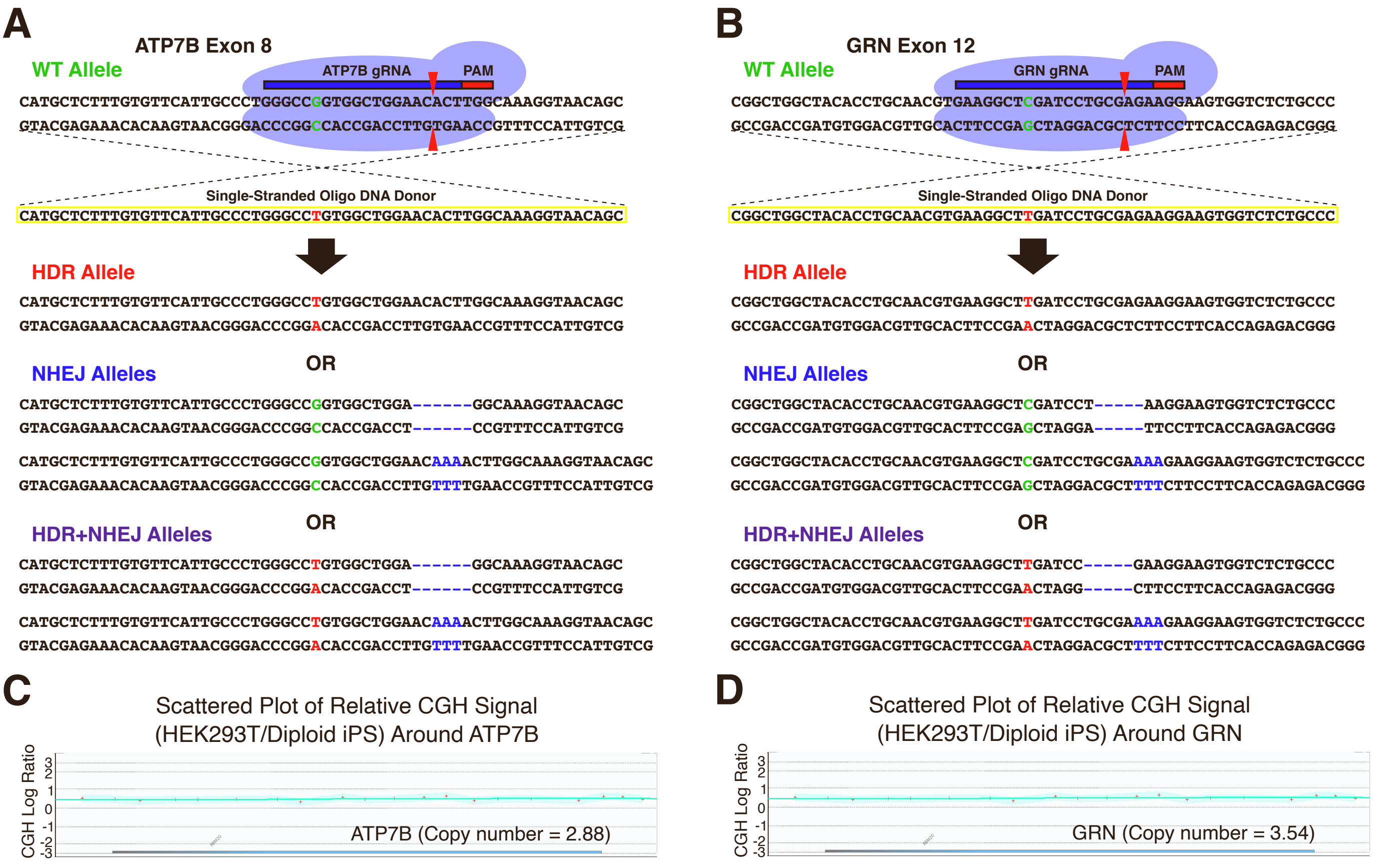

Figure S7. Design of ATP7B R778L and GRN R493X targeting, and estimation of copy numbers of ATP7B and GRN in HEK293T cells, Related to Figure 5.

(A, B) Design of genome editing in ATP7B (A) and GRN (B), respectively. We introduced the R778L (c.2333C>G) (A) and R493X (c.1477C>T) (B) mutations using HypaCas9 and a single-stranded oligonucleotide donor DNA. The resulting HDR alleles have a single nucleotide substitution from C to G (A) and C to T (B), whereas the NHEJ alleles have various insertions and deletions. (C, D) Scattered plots of the relative CGH signal of HEK293T cells in comparison to diploid human iPS cells around the ATP7B (C) and GRN (D) genes, respectively. The relative CGH signals of HEK293T cells in comparison to diploid iPS cells are represented by +. No microduplications or microdeletions were detected around the ATP7B (C) or GRN (D) loci. The median CGH log ratios in these genomic regions shown here were applied to the formula shown in Figure 1E to calculate the copy numbers of ATP7B (C) and GRN (D) were 2.88 (C) and 3.54 (D), respectively.

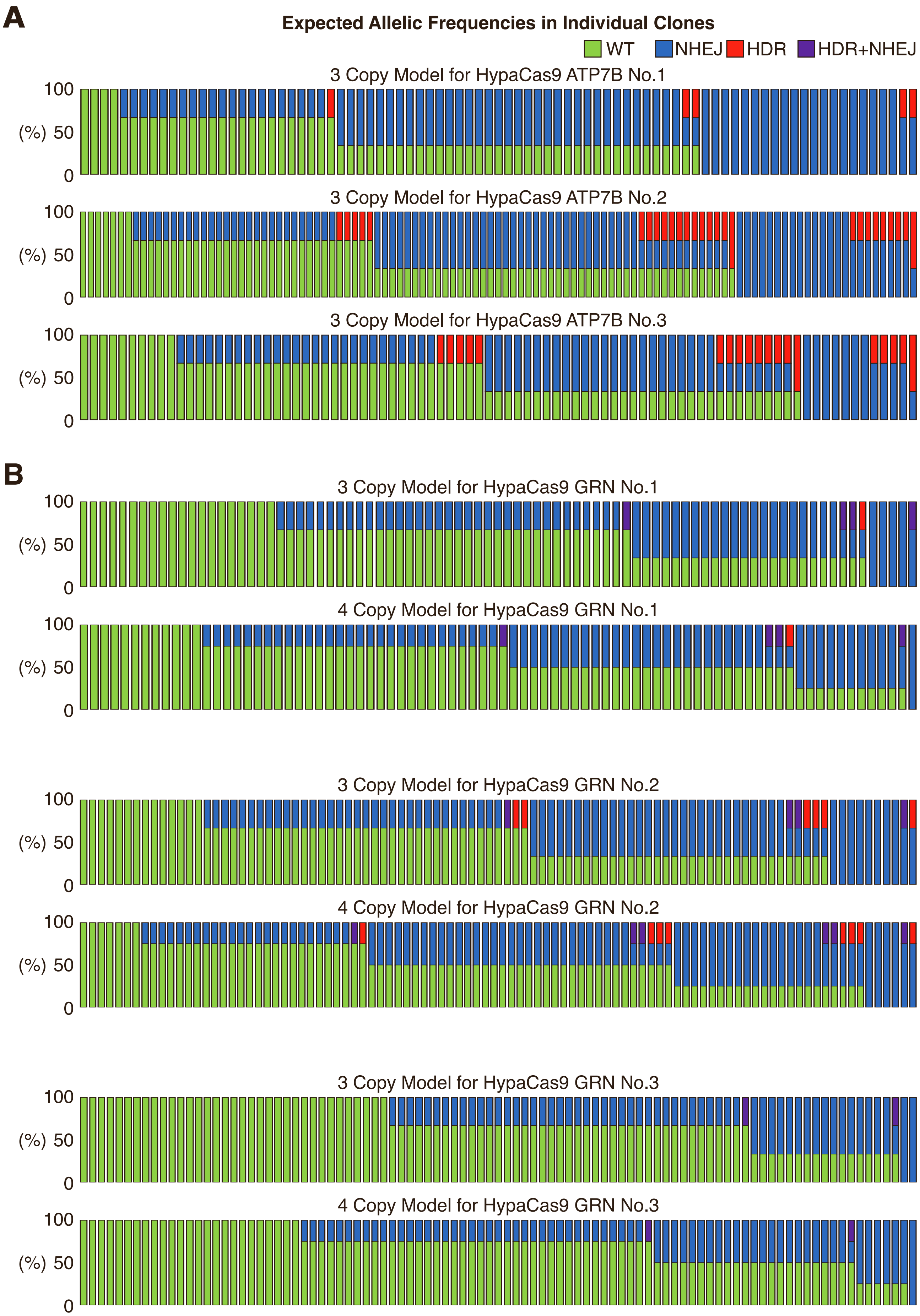
A
Expected Allelic Frequencies in Individual Clones
WT
NHEJ
HDR
HDR+NHEJ
3 Copy Model for HypaCas9 ATP7B No.1
3 Copy Model for HypaCas9 ATP7B No.2
3 Copy Model for HypaCas9 ATP7B No.3
B
3 Copy Model for HypaCas9 GRN No.1
4 Copy Model for HypaCas9 GRN No.1
3 Copy Model for HypaCas9 GRN No.2
4 Copy Model for HypaCas9 GRN No.2
3 Copy Model for HypaCas9 GRN No.3
4 Copy Model for HypaCas9 GRN No.3
100
(%) 50
0

Figure S8. Models assuming random ATP7B R778L and GRN R493X mutagenesis by HypaCas9 in HEK293T cells, Related to Figure 5.

(A-B) Models of the distribution of HEK293T cell clones with different genome editing outcomes by HypaCas9, if genome editing randomly occurred at the overall frequencies in HEK293T cells with three copies of ATP7B (A) and three or four copies of GRN (B) in experiment Nos. 1-3.

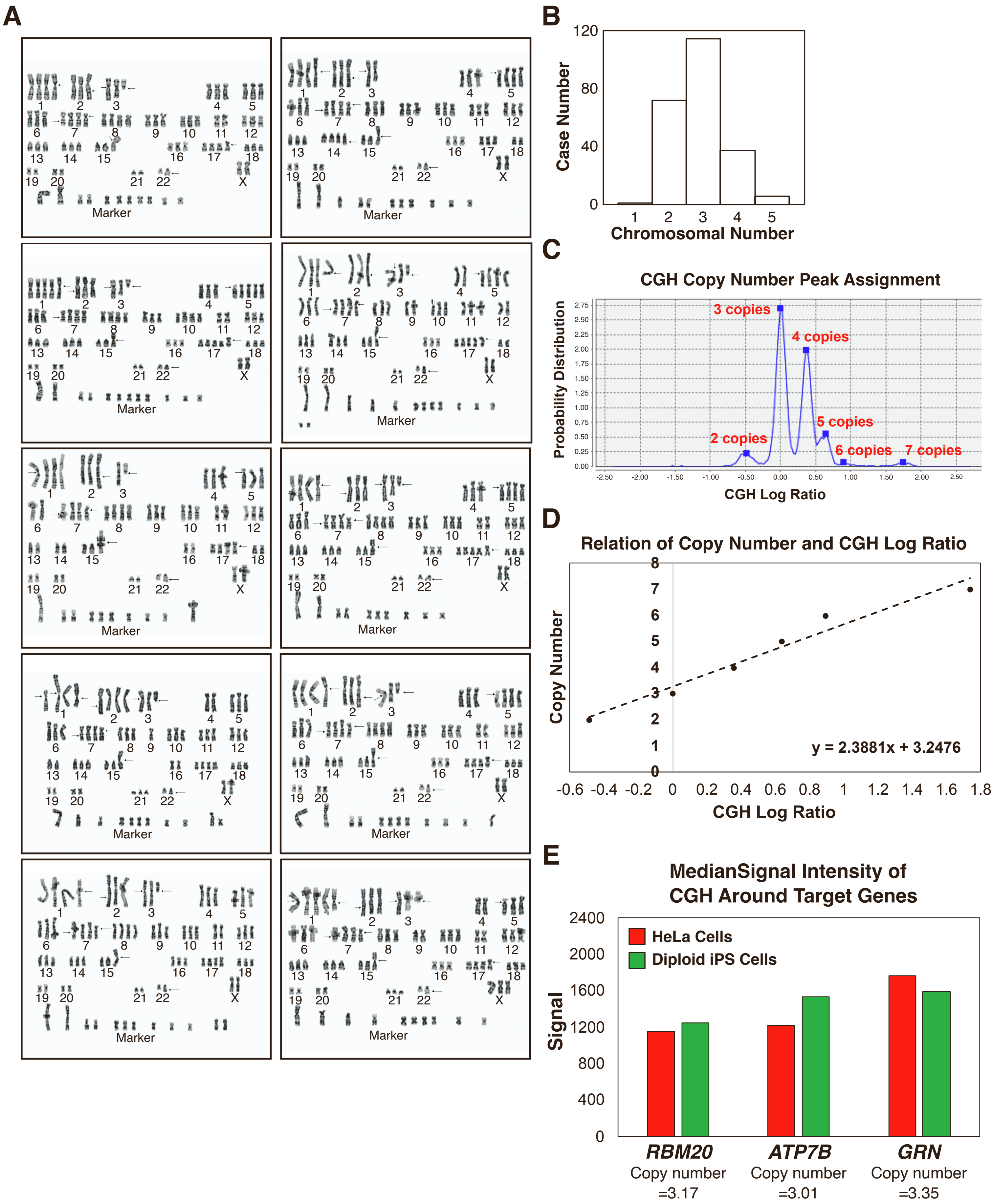
A
Marker
B
Case Number
Chromosomal Number
C
CGH Copy Number Peak Assignment
Probability Distribution
CGH Log Ratio
2 copies
3 copies
4 copies
5 copies
6 copies
7 copies
D
Relation of Copy Number and CGH Log Ratio
Copy Number
CGH Log Ratio
y = 2.3881x + 3.2476
E
MedianSignal Intensity of
CGH Around Target Genes
Signal
HeLa Cells
Diploid iPS Cells
RBM20
Copy number
=3.17
ATP7B
Copy number
=3.01
GRN
Copy number
=3.35

Figure S9. Karyotyping and a CGH analysis to estimate the copy number of RBM20, ATP7B, and GRN in HeLa cells, Related to Figure 6.

(A) Karyotypes of ten HeLa cells. (B) Case numbers observed for each chromosomal number in HEK293T cells. (C) CGH copy number peak assignment. In the CGH analysis, there were several peaks of the CGH signal ratio between HeLa cells and diploid iPS cells based on the chromosomal numbers. The highest peak corresponded to three copies per cell, and the other peaks were incrementally assigned to the other copy numbers. (D) Line of fit between the copy number and the CGH log ratio based on the peak assignment shown in (C). (E) The median signal intensities of CGH around the RBM20, ATP7B, and GRN genes. By applying these values to calculate the CGH log ratios, which were then applied to the formula shown in (D), the copy numbers of RBM20, ATP7B, and GRN, in HeLa cells were 3.17, 3.01, and 3.35, respectively.

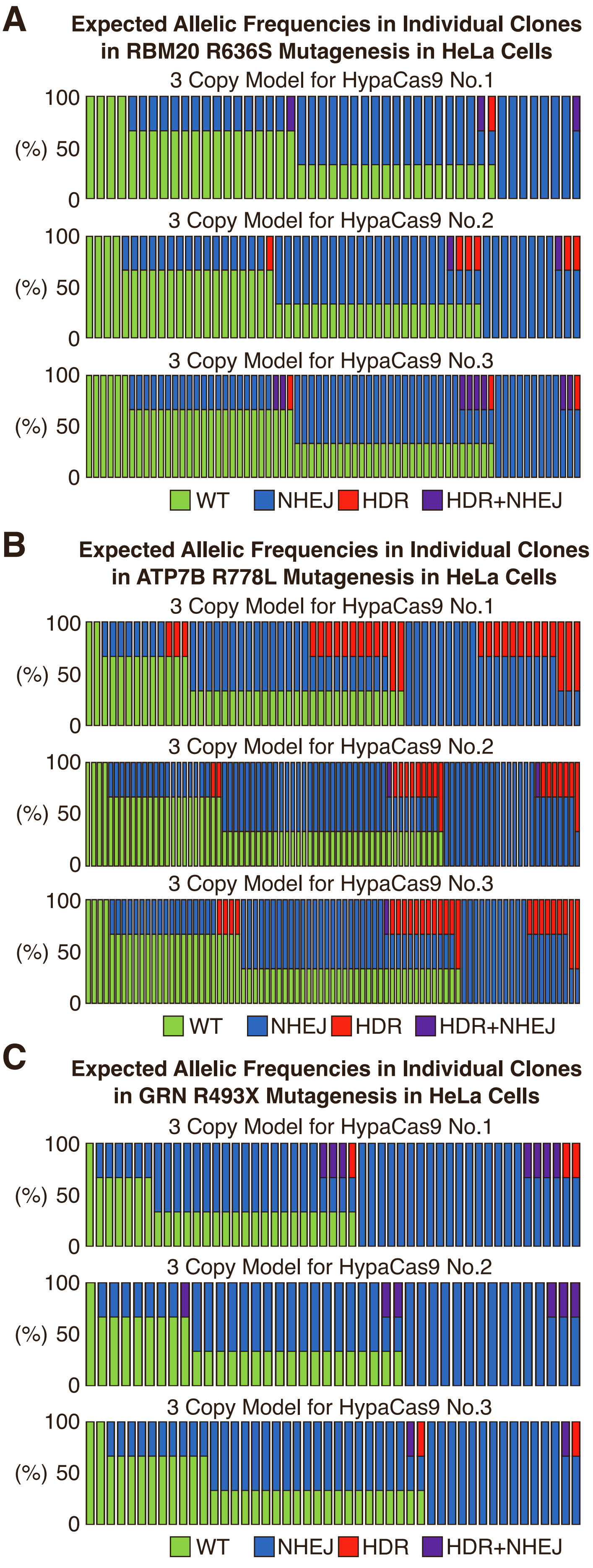

Figure S10. Models assuming random RBM20 R636S, ATP7B R778L, and GRN R493X mutagenesis by HypaCas9 in HeLa cells, Related to Figure 6.

(A-C) Models of the distribution of HeLa cell clones with different genome editing outcomes by HypaCas9, if genome editing randomly occurred at the overall frequencies in HeLa cells with three copies of RBM20 (A), ATP7B (B), and GRN (C) in experiment Nos. 1-3.

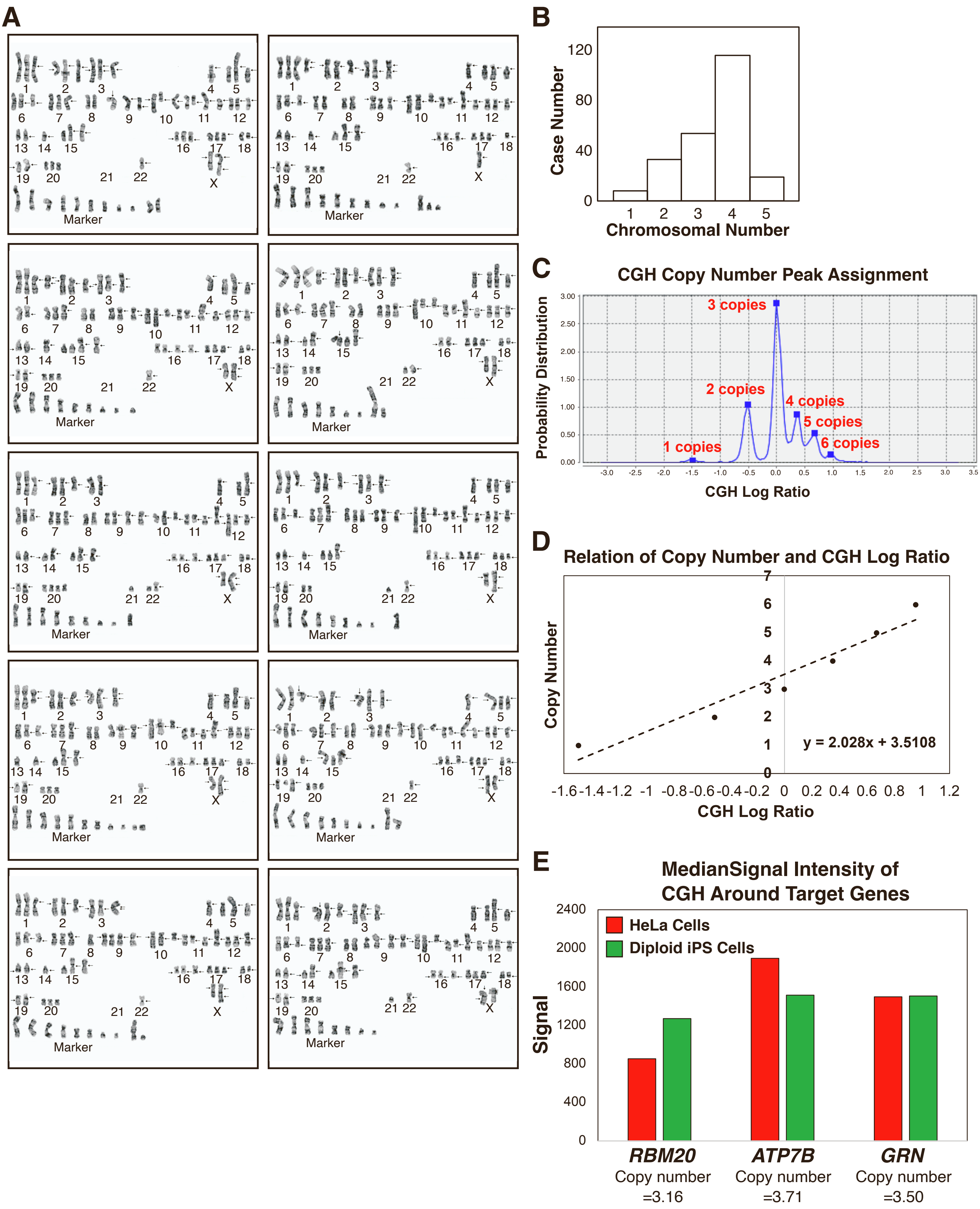
A
Marker
X
B
Case Number
Chromosomal Number
C
CGH Copy Number Peak Assignment
Probability Distribution
CGH Log Ratio
1 copies
2 copies
3 copies
4 copies
5 copies
6 copies
D
Relation of Copy Number and CGH Log Ratio
Copy Number
y = 2.028x + 3.5108
CGH Log Ratio
E
MedianSignal Intensity of
CGH Around Target Genes
HeLa Cells
Diploid iPS Cells
Signal
RBM20
Copy number
=3.16
ATP7B
Copy number
=3.71
GRN
Copy number
=3.50

Figure S11. Karyotyping and a CGH analysis to estimate the copy number of RBM20, ATP7B, and GRN in PC9 cells, Related to Figure 7.

(A) Karyotypes of ten PC9 cells. (B) Case numbers observed for each chromosomal number in HEK293T cells. (C) CGH copy number peak assignment. In the CGH analysis, there were several peaks of the CGH signal ratio between PC9 cells and diploid iPS cells based on the chromosomal numbers. The highest peak corresponded to three copies per cell, and the other peaks were incrementally assigned to the other copy numbers. (D) Line of fit between the copy number and the CGH log ratio based on the peak assignment shown in (C). (E) The median signal intensities of CGH around the RBM20, ATP7B, and GRN genes. By applying these values to calculate the CGH log ratios, which were then applied to the formula shown in (D), the copy numbers of RBM20, ATP7B, and GRN in PC9 cells were 3.16, 3.71, and 3.50, respectively.

A

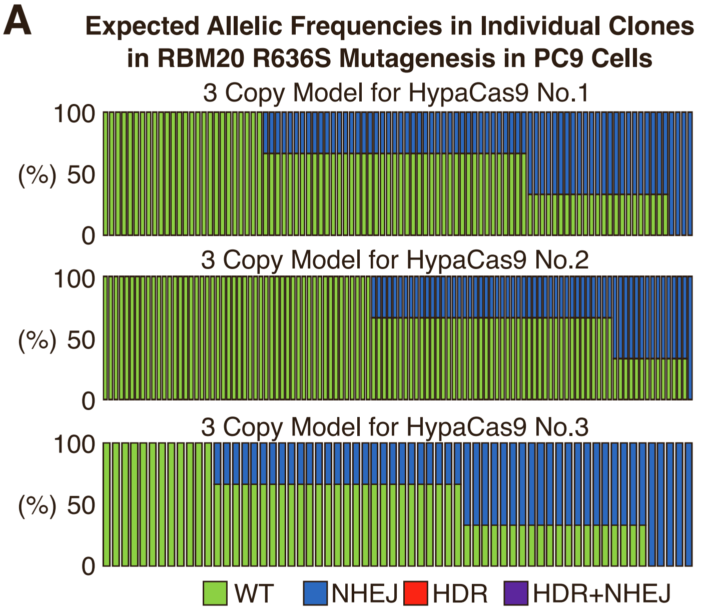

B

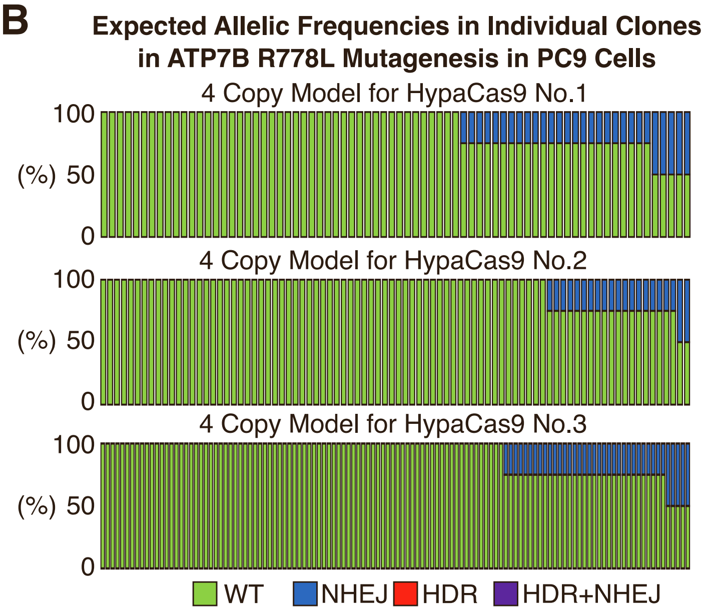

C

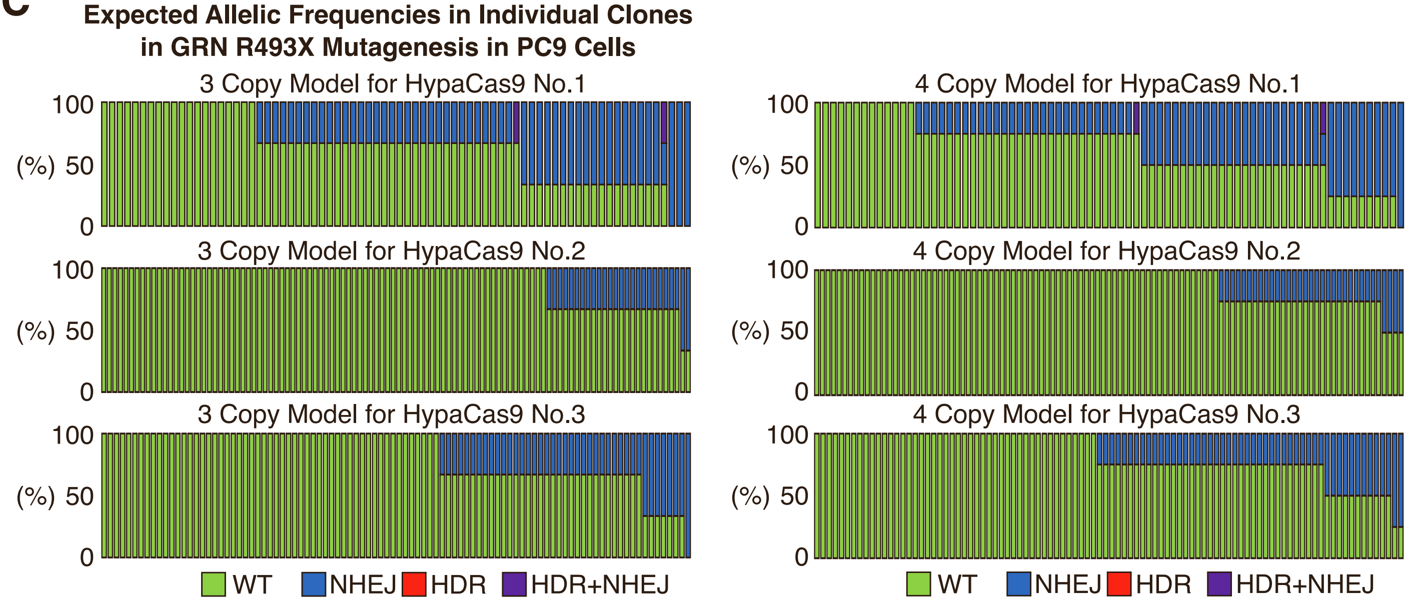

Figure S12. Models assuming random RBM20 R636S, ATP7B R778L, and GRN R493X mutagenesis by HypaCas9 in PC9 cells, Related to Figure 7.

(A-C) Models of the distribution of PC9 cell clones with different genome editing outcomes by HypaCas9, if genome editing randomly occurred at the overall frequencies in PC9 cells with three copies of RBM20 (A), four copies of ATP7B (B), and three or four copies of GRN (C) in experiment Nos. 1-3.

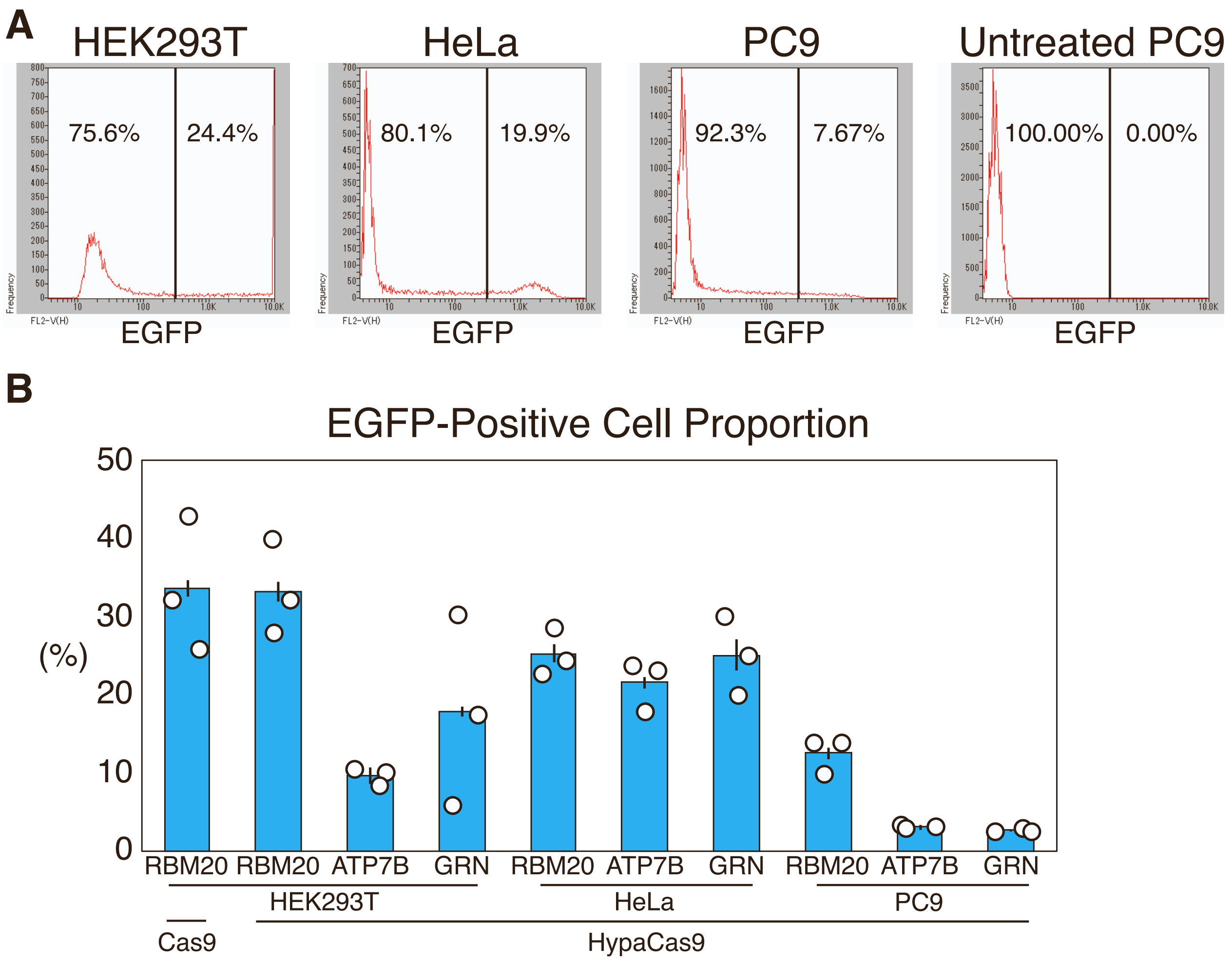

Figure S13. EGFP-positive populations after co-expression of Cas9/HypaCas9, gRNA, and EGFP in HEK293T cells, HeLa cells, and PC9 cells, Related to Figure 2.

(A) Representative histograms of EGFP-positive populations in HEK293T cells, HeLa cells, and PC9 cells 2 days after transfection of the plasmids to target RBM20. PC9 cells without any treatment are shown as a negative cell population. The expression level of EGFP in HEK293T cells was saturated in this histogram. (B) EGFP-positive cell populations determined by flow cytometry. Values ± S.E. are shown (n=3).

Table S1. Chromosomal numbers in HEK293T cells, Related to Figure 3

We counted the chromosomal numbers in ten HEK293T cells. There were 1, 8, 64, 118, 37, and 2 cases where the chromosomal number was 0, 1, 2, 3, 4, and 5, respectively (see also Figure S1B). The most frequent chromosomal number was three. We observed no Y chromosomes, as HEK293T cells were derived from a female. There were no cases where the chromosomal number was more than 6.

#	Cell 1	Cell 2	Cell 3	Cell 4	Cell 5	Cell 6	Cell 7	Cell 8	Cell 9	Cell 10
1	4	3	3	4	3	4	4	4	4	4
2	2	2	2	2	2	3	3	2	4	4
3	3	3	3	3	3	3	3	3	3	3
4	3	3	3	3	3	2	3	3	3	3
5	3	3	3	2	3	2	3	3	3	3
6	3	4	4	4	4	2	2	2	2	2
7	3	3	3	2	3	3	3	3	3	3
8	2	3	3	3	2	3	3	4	3	3
9	2	2	2	2	2	2	2	2	2	2
10	4	3	4	3	4	3	3	4	4	3
11	3	3	3	3	3	3	3	3	3	3
12	3	3	3	3	3	3	3	3	2	4
13	2	1	2	3	2	2	2	1	1	2
14	4	4	3	3	3	3	3	3	3	2
15	1	2	2	0	3	2	3	2	3	2
16	3	3	3	3	3	2	3	3	3	2
17	4	4	3	2	4	4	4	4	4	3
18	2	2	2	2	2	2	2	2	2	2
19	3	3	3	3	3	3	2	2	2	2
20	3	3	3	3	3	3	3	3	2	3
21	3	2	2	1	2	1	1	1	2	2
22	4	3	3	4	4	2	3	3	3	3
X	3	5	4	4	5	4	4	4	3	3
Y	0	0	0	0	0	0	0	0	0	0

Table S2. Calculation of copy numbers of RBM20, ATP7B, and GRN in HEK293T cells, HeLa cells, and PC9 cells, Related to Figures 3, 6, and 7

As shown in Figure 1E, we draw a fitting line between copy number (y-value) and CGH Log Ratio (x-value), which is the ratio of signal intensity between target cells and diploid WTC11 iPS cells. For example, the signal intensity of the RBM20 genomic region for HEK293T cells and WTC11 iPS cells were 1403.986 and 1134.895, respectively. Therefore, the CGH signal ratio was 1403.986/1134.895 = 1.237106516, so the CGH Log Ratio was Log2(1.237106516) = 0.092407095. This is the x-value. As a result, the copy number of RBM20 in HEK293T cells is y = 3.1355*0.09240795 + 3.2572 = 3.546945127.

The signal intensities of the target genomic regions, their ratios, and calculated copy numbers are summarized.

**HEK293T**	Target Intensity	WTC11 Intensity	Target/WTC11	CGH Log Ratio	**Copy Number**
RBM20	1403.986	1134.895	1.237106516	0.092407095	3.55
ATP7B	1172.022	1542.65	0.759745892	-0.11933164	2.88
GRN	1853.811	1510.843	1.227004394	0.088846118	3.54

**Hela**	Target Intensity	WTC11 Intensity	Target/WTC11	CGH Log Ratio	**Copy Number**
RBM20	1158.695	1244.22	0.931262156	-0.030928045	3.17
ATP7B	1217.434	1536.074	0.792562077	-0.100966712	3.01
GRN	1760.424	1588.115	1.10849907	0.044735333	3.35

**PC-9**	Target Intensity	WTC11 Intensity	Target/WTC11	CGH Log Ratio	**Copy Number**
RBM20	850.318	1271.19	0.668914954	-0.174629095	3.16
ATP7B	1893.332	1513.248	1.251170991	0.097316667	3.71
GRN	1490.335	1501.028	0.992876216	-0.003104893	3.50

Table S3. Chromosomal numbers in HeLa cells, Related to Figure 6

We counted the chromosomal numbers in ten HeLa cells. There were 1, 72, 114, 37, and 6 cases where the chromosomal number was 1, 2, 3, 4, and 5, respectively (see also Figure S9B). The most frequent chromosomal number was three. We observed no Y chromosomes, as HeLa cells were derived from a female. There were no cases where the chromosomal number was 0 or more than 6.

#	Cell 1	Cell 2	Cell 3	Cell 4	Cell 5	Cell 6	Cell 7	Cell 8	Cell 9	Cell 10
1	4	5	4	4	4	4	4	4	4	5
2	3	3	3	4	3	3	3	3	2	3
3	3	3	2	3	3	2	3	3	3	3
4	3	3	3	2	3	3	2	3	3	2
5	3	5	3	3	3	4	4	4	4	3
6	3	3	2	3	3	3	3	3	3	3
7	4	4	4	4	4	4	4	4	4	4
8	4	4	4	3	4	3	3	4	4	3
9	3	3	3	1	3	3	2	3	3	2
10	3	4	3	3	3	3	3	3	3	3
11	2	2	2	3	2	3	3	2	2	2
12	3	3	3	3	3	3	2	3	3	3
13	3	3	2	2	3	3	3	3	3	3
14	3	3	2	3	3	4	3	3	3	3
15	3	3	3	3	3	3	3	3	3	3
16	3	3	2	2	3	3	3	3	3	3
17	4	5	4	4	4	3	5	5	4	4
18	3	3	2	3	3	2	2	3	3	3
19	2	2	2	2	2	2	2	2	2	2
20	2	2	2	2	2	2	2	2	2	2
21	2	2	2	3	2	2	2	2	2	3
22	2	2	2	2	2	2	2	2	2	2
X	2	2	2	2	2	2	2	2	2	3
Y	0	0	0	0	0	0	0	0	0	0

Table S4. Chromosomal numbers in PC9 cells, Related to Figure 7

We counted the chromosomal numbers in ten PC9 cells. There were 8, 33, 54, 116, and 19 cases where the chromosomal number was 0, 1, 2, 3, and 4, respectively (see also Figure S11B). The most frequent chromosomal number was three. We observed no Y chromosomes, as PC9 cells were derived from a female. There were no cases where the chromosomal number was more than 5.

#	Cell 1	Cell 2	Cell 3	Cell 4	Cell 5	Cell 6	Cell 7	Cell 8	Cell 9	Cell 10
1	3	4	3	3	3	4	4	3	3	3
2	3	3	3	3	3	3	3	3	2	3
3	3	3	2	3	3	3	3	3	3	3
4	1	1	1	1	1	1	1	1	1	1
5	3	3	2	3	3	3	3	3	3	3
6	3	2	3	3	3	3	3	3	3	3
7	3	3	3	3	3	2	3	3	3	3
8	2	2	2	2	1	2	2	3	2	2
9	3	3	4	3	4	3	3	3	4	4
10	3	3	3	3	3	3	3	3	3	3
11	4	4	4	4	4	4	4	4	4	4
12	3	3	3	3	3	3	3	3	3	3
13	2	2	2	1	2	2	2	2	2	2
14	1	1	2	1	1	1	2	1	1	2
15	3	3	3	3	3	3	3	3	3	3
16	3	1	3	3	3	3	2	3	3	3
17	3	4	0	3	3	3	3	3	3	3
18	2	3	2	2	2	1	2	3	2	2
19	2	2	2	2	2	2	2	2	3	2
20	3	3	4	3	3	3	3	2	3	3
21	0	0	1	0	0	0	0	1	0	1
22	1	1	2	1	1	1	2	1	1	1
X	2	2	2	2	2	1	2	2	2	2
Y	0	0	0	0	0	0	0	0	0	0

Table S5. Commands for the CRISPResso2 analysis used in this study, Related to STAR Methods

CRISPResso version (CRISPResso Batch mode): 2.0.45

Commands used:

/opt/conda/bin/CRISPResso -o /DATA/CRISPRessoBatch_on_batch --name **(File Name 1)** --needleman_wunsch_gap_extend -2 --aln_seed_count 5 --amplicon_name Reference --amplicon_seq **(Sequence 1)** --max_rows_alleles_around_cut_to_plot 50 --prime_editing_pegRNA_extension_quantification_window_size 5 --fastq_r1 **(File Name 2)** --quantification_window_size 1 --quantification_window_center -3 --trimmomatic_command trimmomatic --conversion_nuc_from C --min_bp_quality_or_N 30 --default_min_aln_score 30 --needleman_wunsch_gap_incentive 1 --min_paired_end_reads_overlap 10 --plot_window_size 20 --prime_editing_pegRNA_scaffold_min_match_length 1 --aln_seed_min 2 --aln_seed_len 10 --expected_hdr_amplicon_seq **(Sequence 2)** --needleman_wunsch_gap_open -20 --max_paired_end_reads_overlap 100 --guide_seq **(Sequence 3)** --dsODN **(Sequence 4)** --conversion_nuc_to T --flexiguide_homology 80 --flash_command flash --min_single_bp_quality 30 --exclude_bp_from_left 15 --needleman_wunsch_aln_matrix_loc EDNAFULL --min_average_read_quality 30 --min_frequency_alleles_around_cut_to_plot 0.2 --exclude_bp_from_right 15

File Name 1_Experiment Name

File Name 2_"File name".fastq.gz

Sequence 1_Reference Sequence

Sequence 2_Excepted HDR Sequence

Sequence 3_gRNA Sequence

Sequence 4_Donor Sequence

Table S6. Oligonucleotide donor DNAs used in this study, Related to STAR Methods

Name	Sequence
RBM20 R636S	ACAGATATGGCCCAGAAAGGCCGCGGTCTAGTAGTCCGGTGAGCCGGTCACTCTCCCCGA
ATP7B R778L	CATGCTCTTTGTGTTCATTGCCCTGGGCCTGTGGCTGGAACACTTGGCAAAGGTAACAGC
GRN R493X	CGGCTGGCTACACCTGCAACGTGAAGGCTTGATCCTGCGAGAAGGAAGTGGTCTCTGCCC

Table S7. gRNAs used in this study, Related to STAR Methods

PAM Sequences are underlined. Bold letters indicate the sites of single nucleotide substitutions.

Name	Sequence
RBM20	GGTCT**C**GTAGTCCGGTGAGCCGG
ATP7B	GGGCC**G**GTGGCTGGAACACTTGG
GRN	GAAGGCT**C**GATCCTGCGAGAAGG

Table S8. First PCR primers for amplicon sequencing, Related to STAR Methods

The 3' side of the primer sequences, represented by lowercase letters, are gene-specific. The 5' side of the primers, represented by the uppercase letters, are the common adaptor sequences.

Primer Name	Sequence (5' -> 3')
RBM20_FW-2	TAACTTACGGAGTCGCTCTACGgtgtctgtgtgtgggtggggtggg
RBM20_RV-2	GGATGGGATTCTTTAGGTCCTGagagctgcaggaggtgaagctgggag
ATP7B NGS FW-1	TAACTTACGGAGTCGCTCTACGttattctctggtcatcctggtg
ATP7B NGS RV-2	GGATGGGATTCTTTAGGTCCTGagcagctcttttctgaacctga
GRN-NGS FW1	TAACTTACGGAGTCGCTCTACGataatgccattctgtgctcccttc
GRN-NGS RV1	GGATGGGATTCTTTAGGTCCTGcactccacgtccttcacacc

Table S9. Second PCR primer for amplicon sequencing[37], Related to STAR Methods

Custom P5 index	Custom P7 index	Custom P5 index sequence	Custom P7 index sequence	Custom P5 adapter sequence (5'->3')
P01_PE1.0	P01_PE2.0	CAAGTGTTC	CAAGTGTTC	AATGATACGGCGACCACCGAGATCTACACTCTTTCCCTACACGACGCTCTTCCGATCTNNNNNCAAGTGTTCTAACTTACGGAGTCGCTCTACG
P02_PE1.0	P02_PE2.0	AGGACATTC	AGGACATTC	AATGATACGGCGACCACCGAGATCTACACTCTTTCCCTACACGACGCTCTTCCGATCTNNNNNAGGACATTCTAACTTACGGAGTCGCTCTACG
P03_PE1.0	P03_PE2.0	CACTAATGG	CACTAATGG	AATGATACGGCGACCACCGAGATCTACACTCTTTCCCTACACGACGCTCTTCCGATCTNNNNNCACTAATGGTAACTTACGGAGTCGCTCTACG
P04_PE1.0	P04_PE2.0	AGCCTGATG	AGCCTGATG	AATGATACGGCGACCACCGAGATCTACACTCTTTCCCTACACGACGCTCTTCCGATCTNNNNNAGCCTGATGTAACTTACGGAGTCGCTCTACG
P05_PE1.0	P05_PE2.0	TTACGCTAA	TTACGCTAA	AATGATACGGCGACCACCGAGATCTACACTCTTTCCCTACACGACGCTCTTCCGATCTNNNNNTTACGCTAATAACTTACGGAGTCGCTCTACG
P06_PE1.0	P06_PE2.0	ACTCTCCGT	ACTCTCCGT	AATGATACGGCGACCACCGAGATCTACACTCTTTCCCTACACGACGCTCTTCCGATCTNNNNNACTCTCCGTTAACTTACGGAGTCGCTCTACG
P07_PE1.0	P07_PE2.0	GTCGATGCA	GTCGATGCA	AATGATACGGCGACCACCGAGATCTACACTCTTTCCCTACACGACGCTCTTCCGATCTNNNNNGTCGATGCATAACTTACGGAGTCGCTCTACG
P08_PE1.0	P08_PE2.0	ACGGGAATT	ACGGGAATT	AATGATACGGCGACCACCGAGATCTACACTCTTTCCCTACACGACGCTCTTCCGATCTNNNNNACGGGAATTTAACTTACGGAGTCGCTCTACG
P09_PE1.0	P09_PE2.0	CGCGCCCAG	CGCGCCCAG	AATGATACGGCGACCACCGAGATCTACACTCTTTCCCTACACGACGCTCTTCCGATCTNNNNNCGCGCCCAGTAACTTACGGAGTCGCTCTACG
P10_PE1.0	P10_PE2.0	ACTAGTTTG	ACTAGTTTG	AATGATACGGCGACCACCGAGATCTACACTCTTTCCCTACACGACGCTCTTCCGATCTNNNNNACTAGTTTGTAACTTACGGAGTCGCTCTACG
P11_PE1.0	P11_PE2.0	AGTATTACA	AGTATTACA	AATGATACGGCGACCACCGAGATCTACACTCTTTCCCTACACGACGCTCTTCCGATCTNNNNNAGTATTACATAACTTACGGAGTCGCTCTACG
P12_PE1.0	P12_PE2.0	AGGTTGGGT	AGGTTGGGT	AATGATACGGCGACCACCGAGATCTACACTCTTTCCCTACACGACGCTCTTCCGATCTNNNNNAGGTTGGGTTAACTTACGGAGTCGCTCTACG
P13_PE1.0	P13_PE2.0	GTGAACCGA	GTGAACCGA	AATGATACGGCGACCACCGAGATCTACACTCTTTCCCTACACGACGCTCTTCCGATCTNNNNNGTGAACCGATAACTTACGGAGTCGCTCTACG
P14_PE1.0	P14_PE2.0	GCACAAAAC	GCACAAAAC	AATGATACGGCGACCACCGAGATCTACACTCTTTCCCTACACGACGCTCTTCCGATCTNNNNNGCACAAAACTAACTTACGGAGTCGCTCTACG
P15_PE1.0	P15_PE2.0	CTGTCTTCG	CTGTCTTCG	AATGATACGGCGACCACCGAGATCTACACTCTTTCCCTACACGACGCTCTTCCGATCTNNNNNCTGTCTTCGTAACTTACGGAGTCGCTCTACG
P16_PE1.0	P16_PE2.0	GACGCGACT	GACGCGACT	AATGATACGGCGACCACCGAGATCTACACTCTTTCCCTACACGACGCTCTTCCGATCTNNNNNGACGCGACTTAACTTACGGAGTCGCTCTACG
P17_PE1.0	P17_PE2.0	CAGCCCATA	CAGCCCATA	AATGATACGGCGACCACCGAGATCTACACTCTTTCCCTACACGACGCTCTTCCGATCTNNNNNCAGCCCATATAACTTACGGAGTCGCTCTACG
P18_PE1.0	P18_PE2.0	AGATATCTG	AGATATCTG	AATGATACGGCGACCACCGAGATCTACACTCTTTCCCTACACGACGCTCTTCCGATCTNNNNNAGATATCTGTAACTTACGGAGTCGCTCTACG
P19_PE1.0	P19_PE2.0	AATACGCAC	AATACGCAC	AATGATACGGCGACCACCGAGATCTACACTCTTTCCCTACACGACGCTCTTCCGATCTNNNNNAATACGCACTAACTTACGGAGTCGCTCTACG
P20_PE1.0	P20_PE2.0	TATCGTGCC	TATCGTGCC	AATGATACGGCGACCACCGAGATCTACACTCTTTCCCTACACGACGCTCTTCCGATCTNNNNNTATCGTGCCTAACTTACGGAGTCGCTCTACG
P21_PE1.0	P21_PE2.0	TCCTGGTAT	TCCTGGTAT	AATGATACGGCGACCACCGAGATCTACACTCTTTCCCTACACGACGCTCTTCCGATCTNNNNNTCCTGGTATTAACTTACGGAGTCGCTCTACG
P22_PE1.0	P22_PE2.0	GGCAGAGGA	GGCAGAGGA	AATGATACGGCGACCACCGAGATCTACACTCTTTCCCTACACGACGCTCTTCCGATCTNNNNNGGCAGAGGATAACTTACGGAGTCGCTCTACG
P23_PE1.0	P23_PE2.0	ACAATGGAG	ACAATGGAG	AATGATACGGCGACCACCGAGATCTACACTCTTTCCCTACACGACGCTCTTCCGATCTNNNNNACAATGGAGTAACTTACGGAGTCGCTCTACG
P24_PE1.0	P24_PE2.0	TAGTGGCCA	TAGTGGCCA	AATGATACGGCGACCACCGAGATCTACACTCTTTCCCTACACGACGCTCTTCCGATCTNNNNNTAGTGGCCATAACTTACGGAGTCGCTCTACG
P25_PE1.0	P25_PE2.0	ATCCTATCT	ATCCTATCT	AATGATACGGCGACCACCGAGATCTACACTCTTTCCCTACACGACGCTCTTCCGATCTNNNNNATCCTATCTTAACTTACGGAGTCGCTCTACG
P26_PE1.0	P26_PE2.0	CTGCTTAGC	CTGCTTAGC	AATGATACGGCGACCACCGAGATCTACACTCTTTCCCTACACGACGCTCTTCCGATCTNNNNNCTGCTTAGCTAACTTACGGAGTCGCTCTACG
P27_PE1.0	P27_PE2.0	CGTGGCTGC	CGTGGCTGC	AATGATACGGCGACCACCGAGATCTACACTCTTTCCCTACACGACGCTCTTCCGATCTNNNNNCGTGGCTGCTAACTTACGGAGTCGCTCTACG
P28_PE1.0	P28_PE2.0	GTAACTAAT	GTAACTAAT	AATGATACGGCGACCACCGAGATCTACACTCTTTCCCTACACGACGCTCTTCCGATCTNNNNNGTAACTAATTAACTTACGGAGTCGCTCTACG
P29_PE1.0	P29_PE2.0	GCTTACGAA	GCTTACGAA	AATGATACGGCGACCACCGAGATCTACACTCTTTCCCTACACGACGCTCTTCCGATCTNNNNNGCTTACGAATAACTTACGGAGTCGCTCTACG
P30_PE1.0	P30_PE2.0	GATGCAGTG	GATGCAGTG	AATGATACGGCGACCACCGAGATCTACACTCTTTCCCTACACGACGCTCTTCCGATCTNNNNNGATGCAGTGTAACTTACGGAGTCGCTCTACG
P31_PE1.0	P31_PE2.0	GGGCTACAA	GGGCTACAA	AATGATACGGCGACCACCGAGATCTACACTCTTTCCCTACACGACGCTCTTCCGATCTNNNNNGGGCTACAATAACTTACGGAGTCGCTCTACG
P32_PE1.0	P32_PE2.0	ATAGCACGC	ATAGCACGC	AATGATACGGCGACCACCGAGATCTACACTCTTTCCCTACACGACGCTCTTCCGATCTNNNNNATAGCACGCTAACTTACGGAGTCGCTCTACG
P33_PE1.0	P33_PE2.0	TATAACTCT	TATAACTCT	AATGATACGGCGACCACCGAGATCTACACTCTTTCCCTACACGACGCTCTTCCGATCTNNNNNTATAACTCTTAACTTACGGAGTCGCTCTACG
P34_PE1.0	P34_PE2.0	AAATGCGTT	AAATGCGTT	AATGATACGGCGACCACCGAGATCTACACTCTTTCCCTACACGACGCTCTTCCGATCTNNNNNAAATGCGTTTAACTTACGGAGTCGCTCTACG
P35_PE1.0	P35_PE2.0	CCATCCAGG	CCATCCAGG	AATGATACGGCGACCACCGAGATCTACACTCTTTCCCTACACGACGCTCTTCCGATCTNNNNNCCATCCAGGTAACTTACGGAGTCGCTCTACG
P36_PE1.0	P36_PE2.0	GTCTGCACC	GTCTGCACC	AATGATACGGCGACCACCGAGATCTACACTCTTTCCCTACACGACGCTCTTCCGATCTNNNNNGTCTGCACCTAACTTACGGAGTCGCTCTACG
